# Analysis of the Mechanism of Zhichuanling Oral Liquid in Treating Bronchial Asthma Based on Network Pharmacology

**DOI:** 10.1155/2020/1875980

**Published:** 2020-01-16

**Authors:** Ruiyin Wang, Jiangtao Lin

**Affiliations:** ^1^Graduate School of Beijing University of Chinese Medicine, Beijing, China; ^2^Respiratory and Critical Care Medicine, China-Japan Friendship Hospital, Beijing, China

## Abstract

Zhichuanling oral liquid (ZOL) as a preparation of traditional Chinese medicine is widely used for the treatment of asthma in China; therefore, it is necessary to systematically clarify bioactive chemical ingredients and the mechanism of action of ZOL. Information on ZOL ingredients and asthma-related targets was collected, and we used the latest systematic pharmacological methods to construct protein-protein interaction network and compound-target network and then visualized them. Finally, GO and KEGG pathway enrichment analysis was conducted through the clusterProfiler package in the R software. The results showed that 58 bioactive ingredients and 42 potential targets of ZOL related to asthma were identified, following six important components and nine hub genes screened. Further cluster and enrichment analysis suggested that NF-κB signaling pathway, PI3K/Akt signaling pathway, IL-17 signaling pathway, Toll-like receptor signaling pathway, and TNF signaling pathway might be core pathways of ZOL for asthma. Our work successfully predicted the active ingredients and potential targets of ZOL and provided the explanation for the mechanism of action of ZOL for asthma through the systematic analysis, which suggested that ZOL played a major role in many ways including reducing airway inflammation and inhibiting airway remodeling and mucus secretion. Moreover, ZOL combined with glucocorticoids may have some effects on severe asthma.

## 1. Introduction

Bronchial asthma is a chronic airway inflammatory disease involving a variety of inflammatory cells, inflammatory factors, and structural cells, affecting above 300 million people in the world [1]. In general, the prevalence of asthma is higher in developed countries than in developing countries, which is a serious public health problem in all ages [2, 3]. Current statistics have shown that the prevalence of asthma in adults was estimated to range from 1.24% in China to 21.0% in Australia, while from 3.4% in Albania to 37.6% in Costa Rica in children [4, 5]. Furthermore, asthma has been found to correlate with work loss, miscalculation, anxiety, and depression, as public health and clinical management priorities, which has a great impact on people's life and causes a widespread concern [6].

Inhaled corticosteroids and long-acting *β*-receptor agonists are the first-line therapy in the treatment of asthma. However, some people have poor compliance because of the adverse reactions of glucocorticoids, which leads to uncontrol of asthma in this part of population [7] and repeatedly seeks medical help, wasting large medical resources. Besides, some asthma patients have long-term regular medications and usually have few symptoms, but there are intermittent or mild asthma attacks [8]. Furthermore, a small number of asthma patients, namely, severe asthma, accounting for 5–10% of the world's asthma population, still poorly controlled even with adequate medical treatment, and these patients have a higher burden on the health system due to deterioration of asthma and need of repeated hospitalization or other additional treatment [9].

Traditional Chinese medicine is a comprehensive medicine system with potential utilization value in clinical practice in China for thousands of years, which has the characteristics of multicomponents and multitargets, presenting synergistic effect on many diseases with fewer side effects [10]. Zhichuanling oral liquid (ZOL), mainly composed of Mahuang, Yangjinhua, bitter almond, and Forsythia, has the functions of Xuanfei Pingchuan, relieving cough and removing phlegm. ZOL has obvious antagonistic effects on airway hyperresponsiveness and ventilatory disorder caused by the cholinergic neurotransmitter acetylcholine and mast cell inflammatory mediator histamine, relieves bronchial smooth muscle spasm, and improves lung ventilation [11, 12]. Clinical research studies have shown that ZOL effectively improved clinical symptoms such as cough and shortness of breath in patients with asthma [13–15]. However, the intrinsic mechanism of ZOL in the treatment of asthma has not been elucidated. Given the fact that ZOL contains hundreds of compounds and acts on a variety of cellular targets, it is difficult to systematically study this mechanism using conventional methods. Therefore, new methods and strategies such as network pharmacology are urgently needed to address this problem, which elucidates the synergistic effects and the underlying mechanism of multicomponents and multitargets [16] and provides other possibilities to understand the interactions among active interactions and relevant targets.

In this study, a network pharmacology strategy involving drug-likeness evaluation, oral bioavailability prediction, multiple drug target prediction as well as other network pharmacology techniques was adopted for investigating the mechanism of action underlying the effectiveness of ZOL against asthma.

## 2. Materials and Methods

### 2.1. Chemical Ingredients Database Building

The chemical components of Mahuang, Yangjinhua, bitter almond, and Forsythia were identified from the Traditional Chinese Medicine Systems Pharmacology (TCMSP, http://lsp.nwu.edu.cn/tcmsp.php) database, TCM Database@Taiwan (TCMID, http://tcm.cmu.edu.tw/), and literature. TCMSP is a unique systems pharmacology database of Chinese herbal medicines which captures the herbs, chemicals, targets, and drug-target networks and TCMID is the most comprehensive TCM database in the world.

### 2.2. Pharmacokinetic Prediction

Oral bioavailability (OB) describes the ratio of the amount absorbed into systemic circulation and is a good indicator of the efficiency of oral administration for drug delivery into systemic circulation. Drug-like properties (DL) refers to the physical and chemical properties, such as solubility, stability, and biological properties, which is associated with good clinical efficacy and has considerable indicative effects in the development of new drugs. OB and DL properties of each herb ingredient were collected from the same database. The prediction tool provided by the Molsoft website (http://www.molsoft.com/docking.html) was used when the OB and DL properties of some chemical components had not been found in the database. We set the indexes of OB ≥ 30% and DL ≧ 0.18 as the screening criteria for active components [17, 18]. Briefly, components that satisfied the screening threshold of OB and DL were regarded as the potential components.

### 2.3. The Prediction of Putative Targets of the Ingredients

All the candidate compounds were retrieved in TSMSP Database and PharmMapper Database (http://59.78.96.61/pharmmapper/) to obtain related targets. We downloaded the UniProt table that represented the UniProt Knowledgebase of the potential protein targets that bound with active components. Then, the Retrieve/ID mapping tool in UniProt was used to convert proteins into official symbol formats of gene targets (http://www.uniprot.org/).

### 2.4. The Prediction of Known Therapeutic Targets

Genes associated with asthma were collected from the GeneCards databases (http://www.genecards.org/), the Online Mendelian Inheritance in Man (OMIM) database (http://www.omim.org/), and NCBI (https://www.ncbi.nlm.nih.gov/gene). We searched for the keywords “asthma” with the species limited as “*Homo sapiens*” through the above databases. Finally, we obtained asthma-related genes from 184 targets from OMIM, 607 targets from GeneCards, and 915 targets from the NCBI. After removing duplicates, 1166 genes were collected. Subsequently, asthma-related genes were compared with potential gene targets of active components to obtain the potential target genes of ZOL that played a major role in asthma.

### 2.5. Network Construction

In order to scientifically and reasonably explain the complex relationship between asthma-related compounds and targets, network analysis was carried out. The putative targets of ZOL, the asthma-related targets, and interactional proteins were connected based on the protein-protein interactions (PPI). Then, the drug-compound-target-disease network was constructed and visualized in the Cytoscape software (version 3.7.1, Boston, MA, USA), which illustrated the relationship between the possible target of ZOL and the known target of asthma. The PPI network of ZOL acting on asthma was conducted in the string software (http://string-db.org/cgi/input.pl), with species limited to “*Homo sapiens*” and a confidence score ≥0.7, and the results were saved in TSV format and imported into the Cytoscape software to visualize and analyze the interaction network. We used the Generate style from statistics tool in Cytoscape to set the node size and color settings reflecting the magnitude of the degree and the thickness of the edge reflecting the size of the combined score to obtain the final protein interaction network. “Degree” refers to the number of links to node *i*, while “betweenness centrality” is defined as the number of shortest paths between pairs of nodes passing through node *i*. “Degree” and “betweenness centrality” are commonly used to describe the topological importance of proteins in networks. Therefore, taking the mean of degree and betweenness centrality as the cutoff point, we selected the target with degree and betweenness centrality above the cutoff point as the hub gene, and the pharmacological effects of key targets were analyzed.

### 2.6. GO and KEGG Pathway Enrichment Performance

To cluster the biological functions and clarify the pathways that are involved in putative drug targets, GO function and KEGG signaling pathways were performed using the clusterProfiler package in R software (ver.3.6.0). GO gene enrichment analysis consists of three different categories: biological processes (BP), molecular functions (MF), and cell components (CC). The *P* ≤ 0.05, as the cutoff value, was calculated by the two-side hypergeometric test method to identify enriched GO terms and the localization of the biological and molecular functions of the proteins, which indicated the relative importance of enriched GO terms and pathways.

## 3. Results

### 3.1. Targets Screening of ZOL and Asthma

A total of 95 chemical ingredients of the four herbal medicines in ZOL were retrieved from TCMSP and TCMID and related literature studies, including 28 ingredients in Mahuang, 27 ingredients in Yangjinhua, 17 ingredients in Kuxingren, and 23 ingredients in Forsythia. After eliminating the redundancy, 65 chemical ingredients and 120 corresponding targets of ZOL were obtained, and at the same time, 1166 therapeutic targets for asthma were collected from GeneCards database, NCBI, and OMIM database in this study. It is worth noting that Eciphin (OB = 43.35%, DL = 0.03), *N*-methylephedrine (OB = 63.64%, DL = 0.04), psi-ephedrine (OB = 52.25%, DL = 0.03), and O-benzoyl-L-(+)-pseudoephedrine (OB = 65.17%, DL = 0.13) have a relatively low DL value, but they were included in this study because they were the major pharmacological compounds identified, which were similar to the previous study [19], while amygdalin (OB = 4.42%, DL = 0.61) has a relatively low OB value, but it is the main ingredient of bitter almond. The targets of the components were imported into UniProt, which were converted into official symbol format, and then gene targets were mapped to the disease targets to obtain the ultimate gene targets of ZOL acting on asthma. As a result, 42 gene targets and 58 corresponding antiasthmatic candidate compounds of ZOL were used for further research (Table 1).

### 3.2. Network Construction and Result Analysis

**Figure 1:**
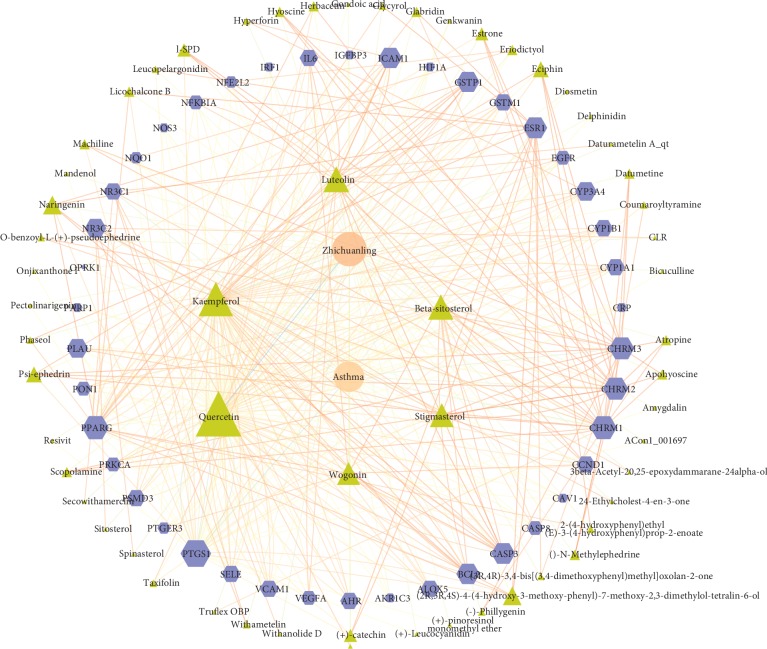
Analysis of the active compounds of zhichuanling and putative asthma targets.

There is no doubt that traditional Chinese medicine plays a therapeutic role through the synergistic effect of various compounds and targets. In order to understand the mechanism of this synergistic effect and the potential mechanism of ZOL in treating asthma, it is essential to understand the effects of these components on the target proteins of asthma. Therefore, we conducted the compounds, the putative target, and asthma-related target network analysis in the Cytoscape 3.7.1 (Figure 1). The results showed that the network consisted of 100 nodes and 420 edges, of which 58 were component nodes and the other 42 were target nodes. In a network, the components or target genes with more degree and betweenness centrality may be the most important on the antiasthmatic effect of ZOL. We analyzed the topology and the six compounds at the top of the degree and betweenness centrality were selected as key components (Table 2).

### 3.3. Construction and Analysis of PPI Network

**Figure 2:**
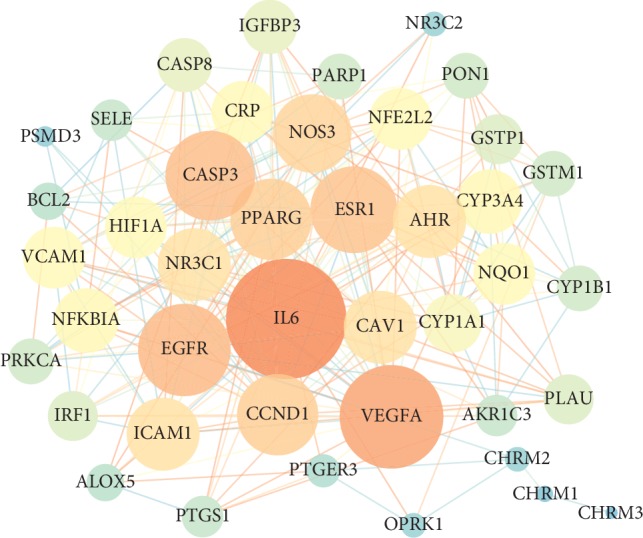
Identification of candidate targets for zhichuanling against asthma.

To further explore the mechanism of ZOL in the treatment of asthma, target genes acting on its corresponding components were submitted to the string software for the construction of PPI network, and high-reliability target proteins interaction data with a score >0.7 were selected. Since protein has little chance of achieving a specified function alone, protein tends to form macromolecular complexes through interaction to complete biological functions in the same cell and the exploration of PPI networks is a viral program to understand cellular tissues, biological processes, and functions. The obtained PPI network file was imported into the Cytoscape software, and the results are shown in Figure 2 after adjusting the parameters. Moreover, we selected out 9 hub genes over the mean of degree and betweenness (Table 3), which were involved in various pathogenic processes of asthma including inflammatory response and immune suppression.

### 3.4. GO and KEGG Pathway Analysis of Target Proteins

**Figure 3:**
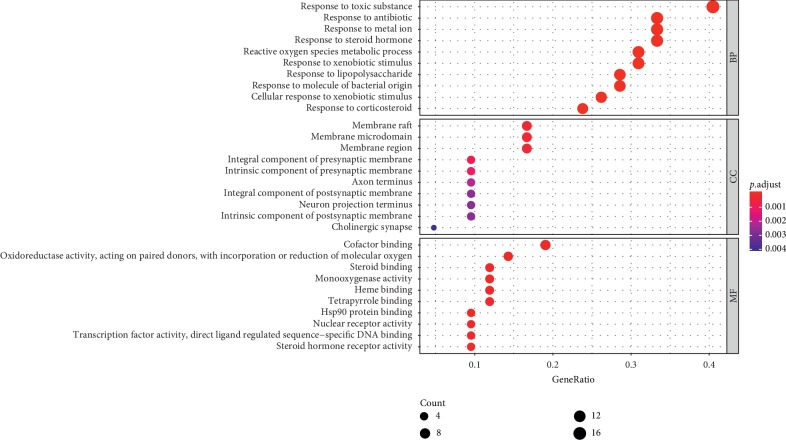
GO analysis of candidate targets for zhichuanling against asthma.

In order to elucidate the biological functions of these genes, GO analysis and KEGG pathway enrichment analysis were carried out in R software based on the clusterProfiler package. GO function was applied to analyze target proteins, and we screened out the top 10 GO entries in BP, MF, and CC (Figure 3). The results indicated that these target proteins were related to response to steroid hormone, reactive oxygen species metabolic process, response to lipopolysaccharide, and response to corticosteroid in BP, cofactor binding, oxidoreductase activity, steroid binding, nuclear receptor activity, transcription factor activity, and steroid hormone receptor activity in MF, and membrane raft, membrane region, integral component of presynaptic and postsynaptic membrane, and intrinsic component of presynaptic and postsynaptic membrane in CC.

**Figure 4:**
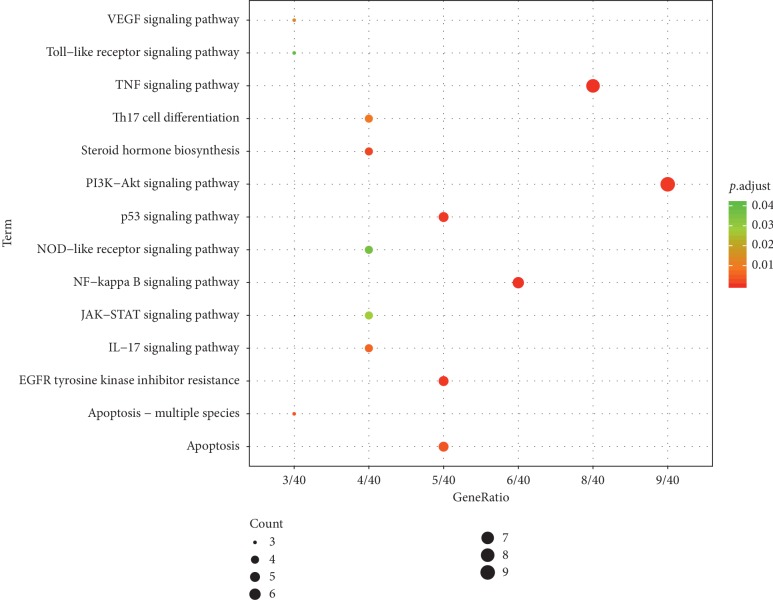
Enrichment analysis of candidate targets for zhichuanling against asthma.

To further confirm that the biological processes associated with target proteins, KEGG pathway analysis was performed to show that these target proteins were enriched in 77 pathways with a *P* value of less than 0.05. We ranked those pathways according to the *P* value of each enriched pathway in an ascending order (Additional [Supplementary-material supplementary-material-1]). The data and biological processes were analyzed to choose the most remarkable relevant significant pathways for further study (Figure 4). From our study results, ZOL possessed multipharmacological effects on asthma involving multipathways.

In these pathways, the most important pathways were PI3K/Akt signaling pathway and NF-*κ*B signaling pathway, which both played an important role in inflammatory response and cell proliferation. Usually, the binding of extracellular cytokines with their corresponding receptors leads to a series of activation of downstream molecules, which activates PI3K/Akt signaling pathway or NF-*κ*B signaling pathway to induce the transcription of related transcription factors and the production of some inflammatory factors in the inflammatory process.

## 4. Discussion

As we all know, traditional Chinese composition, many medicines and ingredients as its character, acts on treating diseases via multiple targets, multiple pathways, and multiple links. Due to the complex composition of natural medicines, its active ingredients are still unclear in clinical and pharmacological research because traditional research methods are difficult to fully clarify its mechanism of action. Nowadays, the rapid development of high-throughput technology, bioinformatics, and network pharmacology technology has effectively solved the problem of multicomponents/multitargets/complex diseases in traditional Chinese medicine. In this study, we are the first to systematically explore the mechanism of action of ZOL on asthma via network pharmacology methods, which can provide direction and insights for subsequent basic and clinical research studies.

Through the network topology analysis, we identified 6 core candidate components of ZOL acting on asthma according to degree and betweenness centrality. Of the 6 components, quercetin had the largest value of degree and betweenness centrality, implicating its critical role in the PPI network, with the function of scavenging free radicals, inhibition of histamine release and interleukin IL-4, and improvement of Th1/Th2 balance, which were often used to treat asthma in the late stage [20]. It is worth noting that the other 5 core components comprising kaempferol, luteolin, *β*-sitosterol, stigmasterol, and wogonin have been studied for their effect on asthma. In general, these components can inhibit airway mucus secretion, reduce the number of eosinophils and relevant cytokine levels including IL-4, IL-5, and IL-13, prevent airway remodeling [21–24], and regulate Th17 pathway [25–27].

We applied the network analysis to screen 9 hub genes including interleukin-6 (IL-6), epidermal growth factor receptor (EGFR), vascular endothelial growth factor A (VEGFA), estrogen receptor 1 (ESR1), cyclin D1 (CCND1), caspase-3 (CASP3), endothelial nitric oxide synthase (NOS3), aryl hydrocarbon receptor (AHR), and peroxisome proliferator-activated receptor gamma (PPARG). From the perspective of network pharmacology, these genes are the key targets for the treatment of asthma at the molecular level, which are linked and constrained to regulate the production and clearance of inflammation and promote cell proliferation and apoptosis. For example, EGFR, a cell surface protein that binds to epidermal growth factor, is expressed in many cells in the lung including smooth muscle cells, endothelial cells, fibroblasts, and epithelial cells [28], and immunoreactivity of which induces mucin production and remodeling of airway tissues [29] and produces airway inflammation through activating intracellular signaling pathways [30, 31]. IL-6, VEGFA, and AHR interact to induce eosinophilic airway inflammation, mucinous metaplasia, subepithelial fibrosis, myocyte proliferation, and dendritic cell activation, leading to airway hyperresponsiveness [32–34] and angiogenesis of airway remodeling [35–38]. However, PPARG and ESR1, as negative regulators, affect the transcription factor signaling pathway by inhibiting NF-*κ*B [39, 40], and some studies have found to support the potential benefits of PPARG agonists in the treatment of asthma. Furthermore, we can see that different components derived from different herbs acted on common targets and the same molecule affected different targets at the same time from the network, indicating that ZOL regulated disease targets through synergistic effects of multiple components.

By the GO enrichment analyses, one interesting phenomenon is observed; that is, ZOL may play an antioxidant and anti-inflammatory role by regulating receptor activity. Nuclear receptors, one of the most abundant transcriptional regulators in animals, have important functions in metabolism, reproductive development, and maintenance of homeostasis. After the cell membrane is externally stimulated, transcription factors in the cytoplasm are activated by phosphorylation or dephosphorylation, enter the nucleus, and are conjunct with the corresponding DNA, and thus, some cytokines such as interleukins and tumor necrosis factors exert their function in oxidative stress by initiating downstream signal transduction. Glucocorticoid insensitivity is the crucial reason leading to severe asthma caused by decreased steroid receptor binding capacity or activity, which may be improved by ZOL from the GO analysis.

It is emphasized on the investigation that the significantly relevant pathways associated with asthma were regarding the anti-inflammatory, cell differentiation, and apoptosis. For example, nuclear factor NF-*κ*B plays a major role in redox-sensitive transcription factors to regulate cytokine activity in airway pathology through a broad spectrum of inflammatory networks. As we all know that glucocorticoids are the most important and effective treatment for asthma, it has a spectral anti-inflammatory effect involved in the inhibition of NF-*κ*B-induced gene transcription. Research studies have been reported that many other antiasthmatic medicines are also developed from the NF-*κ*B signaling pathway. The PI3K-Akt signaling pathway is another important pathway that produces a marked effect in regulating cell survival, proliferation, and apoptosis, which mediates downstream responses under external stimuli such as inflammation and infection. Some experiments have found that the relevant factors are at a high level of expression in the PI3K-Akt signaling pathway in asthma, and airway inflammation and airway remodeling are relieved and reversed after the administration of the inhibitor [41]. Pattern recognition receptors, including Toll-like receptors (TLRs) and nucleotide-binding oligomerization domain-like receptors (NLRs), are able to recognize a variety of common molecular motifs from the microbes and damaged molecules during states of cell stress [42, 43]. Human airway smooth muscle cells are activated by TLR and NLR to promote the development of synthetic phenotypes, enhance the ability of cells to release inflammatory mediators, acquire immunomodulatory properties by interaction with other cells, and reduce contractile state [44]. In addition, Th17 has been considered to be a new type of proinflammatory CD4^+^ T effector cell, which is different from Th1 and Th2 cell lines [45]. Moderate asthma and severe asthma are usually related to the increase of neutrophils and Th17 cytokines such as IL-17A, IL-17F, and IL-22 producing the secretion of epithelial-derived neutrophil chemokines to attract neutrophils in the airways [46]. Moreover, Th17 cytokines also induce mucinous cell metaplasia and airway smooth muscle proliferation to lead to airway stenosis [47, 48]. Finally, TNF, a kind of cytokine with many biological effects, binds to specific receptors on the cell membrane to induce apoptotic pathway, NF-*κ*B pathway, and JNK signaling pathway, achieving cell growth, differentiation, apoptosis, and inflammation, which is involved in the development of asthmatic airway inflammation [49–52]. After given the anti-TNF-*α*, occurrence of asthma may be prevented [53].

## 5. Conclusion

Our study systematically elaborated on the possible mechanisms of ZOL and found that ZOL acted on asthma through multicomponent and multipathway involving airway inflammation, airway remodeling, and mucus secretion. Most importantly, inhaled corticosteroids are the first-line treatment for asthma, but have little effect on neutrophilic inflammation and airway remodeling, while ZOL as add-on may have some effect on the treatment of severe asthma. However, this study has some limitations based on network research and machine algorithms and it is necessary to further verify the antiasthma effect of ZOL through experiments.

## Figures and Tables

**Table 1:** Candidate compounds acting on asthma targets.

ID	Compound	OB (%)	DL
MOL010788	Leucopelargonidin	57.97	0.24
MOL002823	Herbacetin	36.07	0.27
MOL010489	Resivit	30.84	0.27
MOL000422	Kaempferol	41.88	0.24
MOL004798	Delphinidin	40.63	0.28
MOL000098	Quercetin	46.43	0.28
MOL000006	Luteolin	36.16	0.25
MOL000358	Beta-sitosterol	36.91	0.75
MOL000449	Stigmasterol	43.83	0.76
MOL000492	(+)-Catechin	54.83	0.24
MOL001494	Mandenol	42	0.19
MOL001755	24-Ethylcholest-4-en-3-one	36.08	0.76
MOL002881	Diosmetin	31.14	0.27
MOL004328	Naringenin	59.29	0.21
MOL004576	Taxifolin	57.84	0.27
MOL005190	Eriodictyol	71.79	0.24
MOL005573	Genkwanin	37.13	0.24
MOL005842	Pectolinarigenin	41.17	0.3
MOL007214	(+)-Leucocyanidin	37.61	0.27
MOL011319	Truflex OBP	43.74	0.24
MOL010785	*N*-Methylephedrine	63.64	0.04
MOL010786	*O*-Benzoyl-L-(+)-pseudoephedrine	65.17	0.13
MOL006594	Eciphin	43.35	0.03
MOL006637	Psi-ephedrine	52.25	0.03
MOL010921	Estrone	53.56	0.32
MOL000359	Sitosterol	36.91	0.75
MOL005030	Gondoic acid	30.7	0.2
MOL000953	CLR	37.87	0.68
MOL002311	Glycyrol	90.78	0.67
MOL004355	Spinasterol	42.98	0.76
MOL004841	Licochalcone B	76.76	0.19
MOL004908	Glabridin	53.25	0.47
MOL005017	Phaseol	78.77	0.58
MOL007207	Machiline	79.64	0.24
MOL012922	l-SPD	87.35	0.54
MOL001320	Amygdalin	4.42	0.61
MOL000173	Wogonin	30.68	0.23
MOL003283	(2R,3R,4S)-4-(4-Hydroxy-3-methoxy-phenyl)-7-methoxy-2,3-dimethylol-tetralin-6-ol	66.51	0.39
MOL003290	(3R,4R)-3,4-bis[(3,4-Dimethoxyphenyl)methyl]oxolan-2-one	52.3	0.48
MOL003295	(+)-Pinoresinol monomethyl ether	53.08	0.57
MOL003306	ACon1_001697	85.12	0.57
MOL003315	3beta-Acetyl-20,25-epoxydammarane-24alpha-ol	33.07	0.79
MOL003330	(-)-Phillygenin	95.04	0.57
MOL003347	Hyperforin	44.03	0.6
MOL003370	Onjixanthone I	79.16	0.3
MOL000791	Bicuculline	69.67	0.88
MOL011093	Apohyoscine	59.68	0.25
MOL001554	Scopolamine	67.97	0.27
MOL011491	Datumetine	84.74	0.18
MOL011495	Daturametelin A_qt	42.04	0.89
MOL011497	(6R)-6-[(1R)-2-Hydroxy-1-[(8S,9S,10R,13S,14S,17R)-1-keto-10,13-dimethyl-4,7,8,9,11,12,14,15,16,17-decahydrocyclopenta[a]phenanthren-17-yl] ethyl]-4-methyl-3-methylol-5,6-dihydropyran-2-one	53.86	0.9
MOL011519	Hyoscine	49.84	0.27
MOL011531	Secowithamerclin	50.21	0.89
MOL011539	Withametelin	83.59	0.77
MOL011540	Withanolide D	58.29	0.76
MOL005406	Atropine	45.97	0.19
MOL000631	Coumaroyltyramine	112.9	0.2
MOL007923	2-(4-Hydroxyphenyl) ethyl (E)-3-(4-hydroxyphenyl) prop-2-enoate	93.36	0.21

**Table 2:** The main chemical components of ZOL.

SUID	Name	Betweenness centrality	Closeness centrality	Degree	Stress	Topological coefficient	Average shortest path length	Type
120	Quercetin	0.16939094	0.57714286	106	9682	0.0862069	1.73267327	mol
124	Kaempferol	0.064428	0.48792271	58	4918	0.15789474	2.04950495	mol
119	Luteolin	0.02399353	0.4529148	21	1942	0.20062696	2.20792079	mol
118	Beta-sitosterol	0.02044829	0.44888889	19	2044	0.26724138	2.22772277	mol
117	Stigmasterol	0.00769755	0.43722944	13	1162	0.36453202	2.28712871	mol
93	Wogonin	0.01538644	0.44493392	9	1540	0.25095785	2.24752475	mol

**Table 3:** Hub gene by screening.

SUID	Name	Betweenness centrality	Closeness centrality	Degree	Stress	Topological coefficient	Average shortest path length
100	IL-6	0.18253444	0.80392157	34	1248	0.34087481	1.24390244
80	VEGFA	0.06891893	0.71929825	28	618	0.37271062	1.3902439
87	EGFR	0.13827934	0.69491525	24	854	0.39957265	1.43902439
73	CASP3	0.03574063	0.66129032	23	366	0.40022297	1.51219512
89	ESR1	0.04716044	0.65079365	22	426	0.3951049	1.53658537
90	CCND1	0.03846635	0.63076923	20	344	0.41025641	1.58536585
188	PPARG	0.02293555	0.63076923	20	270	0.41794872	1.58536585
84	NOS3	0.02562442	0.62121212	19	280	0.41430499	1.6097561
76	AHR	0.0245402	0.60294118	17	278	0.43589744	1.65853659

## Data Availability

The data used to support the results of this study can be obtained from the corresponding author.

## Supplementary Materials

Supplementary Materials Table S1: 77 KEGG pathways associated with target proteins.

## References

[1] Singh D., Agusti A., Anzueto A. (2019). Global strategy for the diagnosis, management, and prevention of chronic obstructive lung disease: the GOLD science committee report 2019. *European Respiratory Journal*.

[2] GBD 2015 Chronic Respiratory Disease Collaborators (2017). Global, regional, and national deaths, prevalence, disability-adjusted life years, and years lived with disability for chronic obstructive pulmonary disease and asthma, 1990-2015: a systematic analysis for the Global Burden of Disease Study 2015. *The Lancet Respiratory Medicine*.

[3] To T., Stanojevic S., Moores G. (2012). Global asthma prevalence in adults: findings from the cross-sectional world health survey. *BMC Public Health*.

[4] Asher M. I., Montefort S., Björkstén B. (2006). Worldwide time trends in the prevalence of symptoms of asthma, allergic rhinoconjunctivitis, and eczema in childhood: ISAAC Phases One and Three repeat multicountry cross-sectional surveys. *The Lancet*.

[5] Lin J., Wang W., Chen P. (2018). Prevalence and risk factors of asthma in mainland China: the CARE study. *Respiratory Medicine*.

[6] Bourdin A., Bjermer L., Brightling C. (2019). ERS/EAACI Statement on severe exacerbations in asthma in adult: facts, priorities and key research questions. *European Respiratory Journal*.

[7] Baddar S., Jayakrishnan B., Al-Rawas O. A. (2014). Asthma control: importance of compliance and inhaler technique assessments. *Journal of Asthma*.

[8] Ding B., Small M. (2017). Disease burden of mild asthma: findings from a cross-sectional real-world survey. *Advances in Therapy*.

[9] Settipane R. A., Kreindler J. L., Chung Y., Tkacz J. (2019). Evaluating direct costs and productivity losses of asthma patients receiving GINA 4/5 therapy in the US. *Annals of Allergy, Asthma & Immunology*.

[10] Yang Y., Li Y., Wang J. (2017). Systematic investigation ofGinkgo BilobaLeaves for treating cardio-cerebrovascular diseases in an animal model. *ACS Chemical Biology*.

[11] Liu B., Wang X., Xuan Y. (2006). Pharmacodynamic study of zhichuanling oral Liquid in treating bronchial asthma. *Pharmacology and Clinics of Chinese Materia Medica*.

[12] Zhang R. (2006). Experimental study of Xuanfei zhichuanling in treating asthma. *Chinese Medicine Emergency*.

[13] Cao C., Wang A., Ding Y., Jin Y. (2019). Therapeutic effect of Zhichuanling inhalation on children with bronchial asthma. *Journal of Chongqing University of Technology (Natural Science)*.

[14] Jiang W., Jiang L., Wang H. (2019). Clinical observation on zhichuanling in treating 50 cases of acute attack of children with asthma. *Guide of China Medicine*.

[15] Zhang Y., Hu Y., Zhang L., Song W. (2019). Clinical observation of zhichuanling in treating bronchial asthma and asthmatic bronchitis. *Beijing Medical Journal*.

[16] Li S., Zhang B. (2013). Traditional Chinese medicine network pharmacology: theory, methodology and application. *Chinese Journal of Natural Medicines*.

[17] Tao W., Xu X., Wang X. (2013). Network pharmacology-based prediction of the active ingredients and potential targets of Chinese herbal Radix Curcumae formula for application to cardiovascular disease. *Journal of Ethnopharmacology*.

[18] Yue S. J., Liu J., Feng W. W. (2017). System pharmacology-based dissection of the synergistic mechanism of huangqi and huanglian for diabetes mellitus. *Frontiers in Pharmacology*.

[19] Tang F., Tang Q., Tian Y., Fan Q., Huang Y., Tan X. (2015). Network pharmacology-based prediction of the active ingredients and potential targets of Mahuang Fuzi Xixin decoction for application to allergic rhinitis. *Journal of Ethnopharmacology*.

[20] Zhu S., Wang H., Zhang J. (2019). Antiasthmatic activity of quercetin glycosides in neonatal asthmatic rats. *Biotech*.

[21] Antwi A. O., Obiri D. D., Osafo N. (2017). Stigmasterol modulates allergic airway inflammation in Guinea pig model of ovalbumin-induced asthma. *Mediators of Inflammation*.

[22] Jang T. Y., Jung A.-Y., Kyung T.-S., Kim D.-Y., Hwang J.-H., Kim Y. H. (2017). Anti-allergic effect of luteolin in mice with allergic asthma and rhinitis. *Central European Journal of Immunology*.

[23] Park S.-H., Gong J.-H., Choi Y.-J., Kang M.-K., Kim Y.-H., Kang Y.-H. (2015). Kaempferol inhibits endoplasmic reticulum stress-associated mucus hypersecretion in airway epithelial cells and ovalbumin-sensitized mice. *PLoS One*.

[24] Yuk J. E., Woo J. S., Yun C.-Y. (2007). Effects of lactose-*β*-sitosterol and *β*-sitosterol on ovalbumin-induced lung inflammation in actively sensitized mice. *International Immunopharmacology*.

[25] Bui T. T., Piao C. H., Song C. H., Lee C.-H., Shin H. S., Chai O. H. (2017). Baicalein, wogonin, andScutellaria baicalensisethanol extract alleviate ovalbumin-induced allergic airway inflammation and mast cell-mediated anaphylactic shock by regulation of Th1/Th2 imbalance and histamine release. *Anatomy & Cell Biology*.

[26] Lucas C. D., Dorward D. A., Sharma S. (2015). Wogonin induces eosinophil apoptosis and attenuates allergic airway inflammation. *American Journal of Respiratory and Critical Care Medicine*.

[27] Ryu E. K., Kim T.-H., Jang E. J. (2015). Wogonin, a plant flavone from Scutellariae radix, attenuated ovalbumin-induced airway inflammation in mouse model of asthma via the suppression of IL-4/STAT6 signaling. *Journal of Clinical Biochemistry and Nutrition*.

[28] Habibovic A., Hristova M., Heppner D. E. (2016). DUOX1 mediates persistent epithelial EGFR activation, mucous cell metaplasia, and airway remodeling during allergic asthma. *JCI Insight*.

[29] El-Hashim A. Z., Khajah M. A., Renno W. M. (2017). Src-dependent EGFR transactivation regulates lung inflammation via downstream signaling involving ERK1/2, PI3Kdelta/Akt and NFkappaB induction in a murine asthma model. *Scientific Reports*.

[30] Biswas D. K., Iglehart J. D. (2006). Linkage between EGFR family receptors and nuclear factor kappaB (NF-κB) signaling in breast cancer. *Journal of Cellular Physiology*.

[31] Tsuchiya K., Jo T., Takeda N. (2010). EGF receptor activation during allergic sensitization affects IL-6-induced T-cell influx to airways in a rat model of asthma. *European Journal of Immunology*.

[32] Broide D., Lotz M., Cuomo A., Coburn D., Federman E., Wasserman S. (1992). Cytokines in symptomatic asthma airways. *Journal of Allergy and Clinical Immunology*.

[33] Marini M., Vittori E., Hollemborg J., Mattoli S. (1992). Expression of the potent inflammatory cytokines, granulocyte-macrophage-colony-stimulating factor and interleukin-6 and interleukin-8, in bronchial epithelial cells of patients with asthma. *Journal of Allergy and Clinical Immunology*.

[34] Wan Z., Tang Y., Song Q. (2019). Gene polymorphisms in VEGFA and COL2A1 are associated with response to inhaled corticosteroids in children with asthma. *Pharmacogenomics*.

[35] Beamer C. A., Shepherd D. M. (2013). Role of the aryl hydrocarbon receptor (AhR) in lung inflammation. *Seminars in Immunopathology*.

[36] Chiba T., Uchi H., Tsuji G., Gondo H., Moroi Y., Furue M. (2011). Arylhydrocarbon receptor (AhR) activation in airway epithelial cells induces MUC5AC via reactive oxygen species (ROS) production. *Pulmonary Pharmacology & Therapeutics*.

[37] Tsai M.-J., Wang T.-N., Lin Y.-S., Kuo P.-L., Hsu Y.-L., Huang M.-S. (2015). Aryl hydrocarbon receptor agonists upregulate VEGF secretion from bronchial epithelial cells. *Journal of Molecular Medicine*.

[38] Villa M., Crotta S., Dingwell K. S. (2016). The aryl hydrocarbon receptor controls cyclin O to promote epithelial multiciliogenesis. *Nature Communications*.

[39] Dong X., Xu M., Ren Z. (2016). Regulation of CBL and ESR1 expression by microRNA-22-3p, 513a-5p and 625-5p may impact the pathogenesis of dust mite-induced pediatric asthma. *International Journal of Molecular Medicine*.

[40] Zhu T., Zhang W., Feng S.-J., Yu H.-P. (2016). Emodin suppresses LPS-induced inflammation in RAW264.7 cells through a PPARγ-dependent pathway. *International Immunopharmacology*.

[41] Zou W., Ding F., Niu C., Fu Z., Liu S. (2018). Brg1 aggravates airway inflammation in asthma via inhibition of the PI3K/Akt/mTOR pathway. *Biochemical and Biophysical Research Communications*.

[42] Liu D., Rhebergen A. M., Eisenbarth S. C. (2013). Licensing adaptive immunity by NOD-like receptors. *Frontiers in Immunology*.

[43] Takeuchi O., Akira S. (2010). Pattern recognition receptors and inflammation. *Cell*.

[44] Månsson Kvarnhammar A., Tengroth L., Adner M., Cardell L.-O. (2013). Innate immune receptors in human airway smooth muscle cells: activation by TLR1/2, TLR3, TLR4, TLR7 and NOD1 agonists. *PLoS One*.

[45] Zhao Y., Yang J., Gao Y.-d., Guo W. (2010). Th17 immunity in patients with allergic asthma. *International Archives of Allergy and Immunology*.

[46] Wong C. K., Lun S. W. M., Ko F. W. S. (2009). Activation of peripheral Th17 lymphocytes in patients with asthma. *Immunological Investigations*.

[47] Nakagome K., Matsushita S., Nagata M. (2012). Neutrophilic inflammation in severe asthma. *International Archives of Allergy and Immunology*.

[48] Newcomb D. C., Peebles R. S. (2013). Th17-mediated inflammation in asthma. *Current Opinion in Immunology*.

[49] Ghebre M. A., Pang P. H., Diver S. (2018). Biological exacerbation clusters demonstrate asthma and chronic obstructive pulmonary disease overlap with distinct mediator and microbiome profiles. *Journal of Allergy and Clinical Immunology*.

[50] Krusche J., Twardziok M., Rehbach K. (2019). TNFAIP3 is a key player in childhood asthma development and environment-mediated protection. *Journal of Allergy and Clinical Immunology*.

[51] Ravi A., Chowdhury S., Dijkhuis A., Bonta P. I., Sterk P. J., Lutter R. (2019). Neutrophilic inflammation in asthma and defective epithelial translational control. *European Respiratory Journal*.

[52] Vroman H., Bergen I. M., van Hulst J. A. C. (2018). TNF-alpha-induced protein 3 levels in lung dendritic cells instruct TH2 or TH17 cell differentiation in eosinophilic or neutrophilic asthma. *Journal of Allergy and Clinical Immunology*.

[53] Ozkars M. Y., Keskin O., Tokur M. (2018). Comparing the effects of fluticasone, anti-IgE and anti-TNF treatments in a chronic asthma model. *Allergologia et Immunopathologia*.

