# Supplementary material for: Analysis of the Mechanism of Zhichuanling Oral Liquid in Treating Bronchial Asthma Based on Network Pharmacology
Source: Evid Based Complement Alternat Med. 2020 Jan 16;2020:1875980. doi: 10.1155/2020/1875980 (PMC6988691; doi:10.1155/2020/1875980)
Supplement: Supplementary Materials — Table S1: 77 KEGG pathways associated with target proteins. [file 1875980.f1.docx]

Table S1 77 KEGG pathways associated with target proteins

| ID | Description | GeneRatio | BgRatio | pvalue | p.adjust | qvalue | geneID | Count |
| --- | --- | --- | --- | --- | --- | --- | --- | --- |
| hsa04933 | AGE-RAGE signaling pathway in diabetic complications | 10/40 | 100/7867 | 4.31E-11 | 4.94E-09 | 2.41E-09 | BCL2/CASP3/ICAM1/SELE/VCAM1/VEGFA/CCND1/IL6/PRKCA/NOS3 | 10 |
| hsa05418 | Fluid shear stress and atherosclerosis | 11/40 | 139/7867 | 5.25E-11 | 4.94E-09 | 2.41E-09 | BCL2/ICAM1/SELE/VCAM1/GSTP1/GSTM1/VEGFA/CAV1/NOS3/NFE2L2/NQO1 | 11 |
| hsa04668 | TNF signaling pathway | 8/40 | 112/7867 | 6.91E-08 | 4.33E-06 | 2.11E-06 | CASP3/ICAM1/SELE/VCAM1/IL6/NFKBIA/CASP8/IRF1 | 8 |
| hsa05205 | Proteoglycans in cancer | 9/40 | 203/7867 | 5.81E-07 | 2.73E-05 | 1.33E-05 | ESR1/CASP3/PLAU/EGFR/VEGFA/CCND1/PRKCA/HIF1A/CAV1 | 9 |
| hsa05143 | African trypanosomiasis | 5/40 | 37/7867 | 1.02E-06 | 3.24E-05 | 1.58E-05 | ICAM1/SELE/VCAM1/IL6/PRKCA | 5 |
| hsa04066 | HIF-1 signaling pathway | 7/40 | 109/7867 | 1.03E-06 | 3.24E-05 | 1.58E-05 | BCL2/EGFR/VEGFA/IL6/PRKCA/HIF1A/NOS3 | 7 |
| hsa05163 | Human cytomegalovirus infection | 9/40 | 225/7867 | 1.38E-06 | 3.70E-05 | 1.80E-05 | CASP3/EGFR/VEGFA/CCND1/IL6/NFKBIA/CASP8/PRKCA/PTGER3 | 9 |
| hsa05167 | Kaposi sarcoma-associated herpesvirus infection | 8/40 | 186/7867 | 3.38E-06 | 7.94E-05 | 3.87E-05 | CASP3/ICAM1/VEGFA/CCND1/IL6/NFKBIA/CASP8/HIF1A | 8 |
| hsa05169 | Epstein-Barr virus infection | 8/40 | 201/7867 | 6.02E-06 | 0.00012571 | 6.12E-05 | BCL2/CASP3/ICAM1/PSMD3/CCND1/IL6/NFKBIA/CASP8 | 8 |
| hsa05215 | Prostate cancer | 6/40 | 97/7867 | 8.23E-06 | 0.00015469 | 7.54E-05 | BCL2/GSTP1/PLAU/EGFR/CCND1/NFKBIA | 6 |
| hsa04064 | NF-kappa B signaling pathway | 6/40 | 100/7867 | 9.82E-06 | 0.00016775 | 8.17E-05 | BCL2/ICAM1/VCAM1/PLAU/NFKBIA/PARP1 | 6 |
| hsa05416 | Viral myocarditis | 5/40 | 60/7867 | 1.17E-05 | 0.00018299 | 8.91E-05 | CASP3/ICAM1/CCND1/CASP8/CAV1 | 5 |
| hsa05225 | Hepatocellular carcinoma | 7/40 | 168/7867 | 1.84E-05 | 0.00026607 | 0.00012961 | GSTP1/GSTM1/EGFR/CCND1/PRKCA/NFE2L2/NQO1 | 7 |
| hsa04115 | p53 signaling pathway | 5/40 | 72/7867 | 2.86E-05 | 0.00038362 | 0.00018687 | BCL2/CASP3/CCND1/CASP8/IGFBP3 | 5 |
| hsa01524 | Platinum drug resistance | 5/40 | 73/7867 | 3.06E-05 | 0.00038362 | 0.00018687 | BCL2/CASP3/GSTP1/GSTM1/CASP8 | 5 |
| hsa00980 | Metabolism of xenobiotics by cytochrome P450 | 5/40 | 76/7867 | 3.72E-05 | 0.00043745 | 0.00021309 | CYP3A4/CYP1A1/CYP1B1/GSTP1/GSTM1 | 5 |
| hsa01521 | EGFR tyrosine kinase inhibitor resistance | 5/40 | 79/7867 | 4.49E-05 | 0.00047022 | 0.00022905 | BCL2/EGFR/VEGFA/IL6/PRKCA | 5 |
| hsa04020 | Calcium signaling pathway | 7/40 | 193/7867 | 4.50E-05 | 0.00047022 | 0.00022905 | CHRM3/CHRM1/CHRM2/EGFR/PRKCA/PTGER3/NOS3 | 7 |
| hsa05204 | Chemical carcinogenesis | 5/40 | 82/7867 | 5.38E-05 | 0.00050834 | 0.00024762 | CYP3A4/CYP1A1/CYP1B1/GSTP1/GSTM1 | 5 |
| hsa04151 | PI3K-Akt signaling pathway | 9/40 | 354/7867 | 5.41E-05 | 0.00050834 | 0.00024762 | CHRM1/CHRM2/BCL2/EGFR/VEGFA/CCND1/IL6/PRKCA/NOS3 | 9 |
| hsa05162 | Measles | 6/40 | 138/7867 | 6.14E-05 | 0.00054972 | 0.00026778 | BCL2/CASP3/CCND1/IL6/NFKBIA/CASP8 | 6 |
| hsa04913 | Ovarian steroidogenesis | 4/40 | 49/7867 | 0.00010296 | 0.00082546 | 0.0004021 | CYP1A1/CYP1B1/ALOX5/AKR1C3 | 4 |
| hsa05144 | Malaria | 4/40 | 49/7867 | 0.00010296 | 0.00082546 | 0.0004021 | ICAM1/SELE/VCAM1/IL6 | 4 |
| hsa05206 | MicroRNAs in cancer | 8/40 | 299/7867 | 0.00010538 | 0.00082546 | 0.0004021 | BCL2/CASP3/CYP1B1/PLAU/EGFR/VEGFA/CCND1/PRKCA | 8 |
| hsa05161 | Hepatitis B | 6/40 | 163/7867 | 0.00015452 | 0.001162 | 0.00056604 | BCL2/CASP3/IL6/NFKBIA/CASP8/PRKCA | 6 |
| hsa05134 | Legionellosis | 4/40 | 55/7867 | 0.00016213 | 0.00117231 | 0.00057106 | CASP3/IL6/NFKBIA/CASP8 | 4 |
| hsa05164 | Influenza A | 6/40 | 167/7867 | 0.00017647 | 0.00122873 | 0.00059854 | CASP3/ICAM1/IL6/NFKBIA/CASP8/PRKCA | 6 |
| hsa00140 | Steroid hormone biosynthesis | 4/40 | 60/7867 | 0.0002276 | 0.0015282 | 0.00074442 | CYP3A4/CYP1A1/CYP1B1/AKR1C3 | 4 |
| hsa04725 | Cholinergic synapse | 5/40 | 112/7867 | 0.0002365 | 0.00153314 | 0.00074683 | CHRM3/CHRM1/CHRM2/BCL2/PRKCA | 5 |
| hsa05145 | Toxoplasmosis | 5/40 | 113/7867 | 0.00024653 | 0.0015449 | 0.00075255 | BCL2/CASP3/ALOX5/NFKBIA/CASP8 | 5 |
| hsa04510 | Focal adhesion | 6/40 | 199/7867 | 0.00045501 | 0.00275943 | 0.00134418 | BCL2/EGFR/VEGFA/CCND1/PRKCA/CAV1 | 6 |
| hsa04926 | Relaxin signaling pathway | 5/40 | 130/7867 | 0.00047197 | 0.00277283 | 0.00135071 | EGFR/VEGFA/NFKBIA/PRKCA/NOS3 | 5 |
| hsa04210 | Apoptosis | 5/40 | 136/7867 | 0.00058039 | 0.00330548 | 0.00161017 | BCL2/CASP3/NFKBIA/CASP8/PARP1 | 5 |
| hsa04215 | Apoptosis - multiple species | 3/40 | 33/7867 | 0.0005978 | 0.00330548 | 0.00161017 | BCL2/CASP3/CASP8 | 3 |
| hsa05210 | Colorectal cancer | 4/40 | 86/7867 | 0.0009013 | 0.00484125 | 0.00235828 | BCL2/CASP3/EGFR/CCND1 | 4 |
| hsa05160 | Hepatitis C | 5/40 | 155/7867 | 0.00104964 | 0.00548146 | 0.00267014 | CASP3/EGFR/CCND1/NFKBIA/CASP8 | 5 |
| hsa05219 | Bladder cancer | 3/40 | 41/7867 | 0.00113557 | 0.00576993 | 0.00281066 | EGFR/VEGFA/CCND1 | 3 |
| hsa04657 | IL-17 signaling pathway | 4/40 | 93/7867 | 0.00120794 | 0.00582289 | 0.00283646 | CASP3/IL6/NFKBIA/CASP8 | 4 |
| hsa05222 | Small cell lung cancer | 4/40 | 93/7867 | 0.00120794 | 0.00582289 | 0.00283646 | BCL2/CASP3/CCND1/NFKBIA | 4 |
| hsa01522 | Endocrine resistance | 4/40 | 98/7867 | 0.00146742 | 0.00689689 | 0.00335963 | ESR1/BCL2/EGFR/CCND1 | 4 |
| hsa04625 | C-type lectin receptor signaling pathway | 4/40 | 104/7867 | 0.00182745 | 0.00837952 | 0.00408185 | IL6/NFKBIA/CASP8/IRF1 | 4 |
| hsa04659 | Th17 cell differentiation | 4/40 | 107/7867 | 0.00202867 | 0.00908072 | 0.00442342 | AHR/IL6/NFKBIA/HIF1A | 4 |
| hsa04726 | Serotonergic synapse | 4/40 | 115/7867 | 0.00263954 | 0.01154029 | 0.00562153 | PTGS1/CASP3/ALOX5/PRKCA | 4 |
| hsa04919 | Thyroid hormone signaling pathway | 4/40 | 119/7867 | 0.00298791 | 0.01276654 | 0.00621887 | ESR1/CCND1/PRKCA/HIF1A | 4 |
| hsa04370 | VEGF signaling pathway | 3/40 | 59/7867 | 0.00325093 | 0.01358168 | 0.00661594 | VEGFA/PRKCA/NOS3 | 3 |
| hsa00590 | Arachidonic acid metabolism | 3/40 | 63/7867 | 0.00391584 | 0.01600387 | 0.00779584 | PTGS1/ALOX5/AKR1C3 | 3 |
| hsa05170 | Human immunodeficiency virus 1 infection | 5/40 | 212/7867 | 0.00413865 | 0.01655461 | 0.00806412 | BCL2/CASP3/NFKBIA/CASP8/PRKCA | 5 |
| hsa05223 | Non-small cell lung cancer | 3/40 | 66/7867 | 0.00446514 | 0.01748845 | 0.00851901 | EGFR/CCND1/PRKCA | 3 |
| hsa04915 | Estrogen signaling pathway | 4/40 | 138/7867 | 0.00507821 | 0.01941589 | 0.00945791 | ESR1/BCL2/EGFR/NOS3 | 4 |
| hsa04917 | Prolactin signaling pathway | 3/40 | 70/7867 | 0.00526708 | 0.01941589 | 0.00945791 | ESR1/CCND1/IRF1 | 3 |
| hsa05120 | Epithelial cell signaling in Helicobacter pylori infection | 3/40 | 70/7867 | 0.00526708 | 0.01941589 | 0.00945791 | CASP3/EGFR/NFKBIA | 3 |
| hsa00982 | Drug metabolism - cytochrome P450 | 3/40 | 72/7867 | 0.00569852 | 0.02060234 | 0.01003585 | CYP3A4/GSTP1/GSTM1 | 3 |
| hsa05165 | Human papillomavirus infection | 6/40 | 330/7867 | 0.00597913 | 0.02120901 | 0.01033138 | CASP3/EGFR/VEGFA/CCND1/CASP8/IRF1 | 6 |
| hsa05212 | Pancreatic cancer | 3/40 | 75/7867 | 0.00638454 | 0.02182354 | 0.01063073 | EGFR/VEGFA/CCND1 | 3 |
| hsa05214 | Glioma | 3/40 | 75/7867 | 0.00638454 | 0.02182354 | 0.01063073 | EGFR/CCND1/PRKCA | 3 |
| hsa05133 | Pertussis | 3/40 | 76/7867 | 0.00662371 | 0.02212197 | 0.0107761 | CASP3/IL6/IRF1 | 3 |
| hsa04080 | Neuroactive ligand-receptor interaction | 6/40 | 338/7867 | 0.00670719 | 0.02212197 | 0.0107761 | CHRM3/CHRM1/CHRM2/PTGER3/OPRK1/NR3C1 | 6 |
| hsa04921 | Oxytocin signaling pathway | 4/40 | 153/7867 | 0.00729696 | 0.023494 | 0.01144445 | EGFR/CCND1/PRKCA/NOS3 | 4 |
| hsa00983 | Drug metabolism - other enzymes | 3/40 | 79/7867 | 0.00737312 | 0.023494 | 0.01144445 | CYP3A4/GSTP1/GSTM1 | 3 |
| hsa04630 | JAK-STAT signaling pathway | 4/40 | 162/7867 | 0.00889591 | 0.02787384 | 0.01357796 | BCL2/EGFR/CCND1/IL6 | 4 |
| hsa05235 | PD-L1 expression and PD-1 checkpoint pathway in cancer | 3/40 | 89/7867 | 0.0102249 | 0.03151282 | 0.01535059 | EGFR/NFKBIA/HIF1A | 3 |
| hsa05323 | Rheumatoid arthritis | 3/40 | 91/7867 | 0.010862 | 0.03293639 | 0.01604404 | ICAM1/VEGFA/IL6 | 3 |
| hsa05146 | Amoebiasis | 3/40 | 95/7867 | 0.01220423 | 0.03605977 | 0.01756551 | CASP3/IL6/PRKCA | 3 |
| hsa04621 | NOD-like receptor signaling pathway | 4/40 | 178/7867 | 0.01227567 | 0.03605977 | 0.01756551 | BCL2/IL6/NFKBIA/CASP8 | 4 |
| hsa05152 | Tuberculosis | 4/40 | 179/7867 | 0.01251077 | 0.03618501 | 0.01762652 | BCL2/CASP3/IL6/CASP8 | 4 |
| hsa05231 | Choline metabolism in cancer | 3/40 | 99/7867 | 0.01363808 | 0.03884787 | 0.01892365 | EGFR/PRKCA/HIF1A | 3 |
| hsa05202 | Transcriptional misregulation in cancer | 4/40 | 186/7867 | 0.01423808 | 0.03995164 | 0.01946132 | PPARG/PLAU/IL6/IGFBP3 | 4 |
| hsa04960 | Aldosterone-regulated sodium reabsorption | 2/40 | 37/7867 | 0.01500576 | 0.04072743 | 0.01983923 | PRKCA/NR3C2 | 2 |
| hsa05216 | Thyroid cancer | 2/40 | 37/7867 | 0.01500576 | 0.04072743 | 0.01983923 | PPARG/CCND1 | 2 |
| hsa05142 | Chagas disease (American trypanosomiasis) | 3/40 | 103/7867 | 0.01516447 | 0.04072743 | 0.01983923 | IL6/NFKBIA/CASP8 | 3 |
| hsa04620 | Toll-like receptor signaling pathway | 3/40 | 104/7867 | 0.01556062 | 0.04120277 | 0.02007078 | IL6/NFKBIA/CASP8 | 3 |
| hsa04928 | Parathyroid hormone synthesis, secretion and action | 3/40 | 106/7867 | 0.01637046 | 0.04274508 | 0.02082207 | BCL2/EGFR/PRKCA | 3 |
| hsa04931 | Insulin resistance | 3/40 | 108/7867 | 0.01720374 | 0.04430551 | 0.02158219 | IL6/NFKBIA/NOS3 | 3 |
| hsa05203 | Viral carcinogenesis | 4/40 | 201/7867 | 0.01843464 | 0.04683394 | 0.02281385 | CASP3/CCND1/NFKBIA/CASP8 | 4 |
| hsa04670 | Leukocyte transendothelial migration | 3/40 | 112/7867 | 0.01894089 | 0.04722887 | 0.02300622 | ICAM1/VCAM1/PRKCA | 3 |
| hsa00380 | Tryptophan metabolism | 2/40 | 42/7867 | 0.01909252 | 0.04722887 | 0.02300622 | CYP1A1/CYP1B1 | 2 |
